# Barriers and Enablers for Implementation of an Artificial Intelligence–Based Decision Support Tool to Reduce the Risk of Readmission of Patients With Heart Failure: Stakeholder Interviews

**DOI:** 10.2196/47335

**Published:** 2023-08-23

**Authors:** Monika Nair, Jonas Andersson, Jens M Nygren, Lina E Lundgren

**Affiliations:** 1 School of Health and Welfare Halmstad University Halmstad Sweden; 2 Cambio Healthcare Systems AB Stockholm Sweden; 3 School of Business, Innovation and Sustainability Halmstad University Halmstad Sweden

**Keywords:** implementation, AI systems, health care, interviews, artificial Intelligence, AI, decision support tool, readmission, prediction, heart failure, digital tool

## Abstract

**Background:**

Artificial intelligence (AI) applications in health care are expected to provide value for health care organizations, professionals, and patients. However, the implementation of such systems should be carefully planned and organized in order to ensure quality, safety, and acceptance. The gathered view of different stakeholders is a great source of information to understand the barriers and enablers for implementation in a specific context.

**Objective:**

This study aimed to understand the context and stakeholder perspectives related to the future implementation of a clinical decision support system for predicting readmissions of patients with heart failure. The study was part of a larger project involving model development, interface design, and implementation planning of the system.

**Methods:**

Interviews were held with 12 stakeholders from the regional and municipal health care organizations to gather their views on the potential effects implementation of such a decision support system could have as well as barriers and enablers for implementation. Data were analyzed based on the categories defined in the nonadoption, abandonment, scale-up, spread, sustainability (NASSS) framework.

**Results:**

Stakeholders had in general a positive attitude and curiosity toward AI-based decision support systems, and mentioned several barriers and enablers based on the experiences of previous implementations of information technology systems. Central aspects to consider for the proposed clinical decision support system were design aspects, access to information throughout the care process, and integration into the clinical workflow. The implementation of such a system could lead to a number of effects related to both clinical outcomes as well as resource allocation, which are all important to address in the planning of implementation. Stakeholders saw, however, value in several aspects of implementing such system, emphasizing the increased quality of life for those patients who can avoid being hospitalized.

**Conclusions:**

Several ideas were put forward on how the proposed AI system would potentially affect and provide value for patients, professionals, and the organization, and implementation aspects were important parts of that. A successful system can help clinicians to prioritize the need for different types of treatments but also be used for planning purposes within the hospital. However, the system needs not only technological and clinical precision but also a carefully planned implementation process. Such a process should take into consideration the aspects related to all the categories in the NASSS framework. This study further highlighted the importance to study stakeholder needs early in the process of development, design, and implementation of decision support systems, as the data revealed new information on the potential use of the system and the placement of the application in the care process.

## Introduction

Artificial intelligence (AI) is expected to add new dimensions of value to patients, professionals, and health care organizations [1,2], and several successful instances of AI use in practice have been reported [3-5]. However, reaching the sustainable use of AI applications is complex, and AI technology is often met with skepticism related to algorithmic and data bias; risks and safety; liability, legal and ethical issues; or concerns about professional roles [6]. Hence, the implementation of AI applications in health care should be carefully planned and organized in order to ensure quality, safety, and acceptance [7]. However, few studies have investigated stakeholders’ views on implementing AI in practice [8,9].

Literature suggests using stakeholder analysis and studying their requirements and concerns early in the process to understand the context in which to implement an innovation, inform the planning process, and identify enablers and barriers for implementation strategy development [10]. Since skepticism toward the introduction and use of AI applications in health care is closely associated with views and understandings held by health care professionals, their involvement and the inclusion of their perspectives in implementation processes are crucial for the initiation and development of such processes. Research on stakeholders’ perspectives regarding an AI application not only informs a particular technological area but will also contribute to grounding the implementation process in the contextual and situational factors that will determine the outcomes from the intended implementation of the technology [11,12]. A more developed understanding of stakeholders’ perspectives will also be important to lay the foundation for the development of theoretical models and frameworks for AI implementation [7].

This study aimed to specifically understand the context and stakeholder perspectives in relation to the implementation of an AI-based decision support application for predicting readmissions of patients with congestive heart failure (HF). This study also contributes to the general body of knowledge about health care professional stakeholder perspectives and potential barriers and enablers in relation to studies and planning of future AI-based implementations of decision support systems in health care.

## Methods

This study had its starting point in the development of an AI-based application for predicting readmissions of patients with HF within 30 days of discharge [13]. To further develop the application and prepare for implementation in a clinical setting, this study was performed to identify important aspects for further development, its potential use in practice, and potential barriers and facilitating factors for the implementation of such a system [10].

### Participants

Data were collected through stakeholder interviews (N=12) in a Swedish health care organization consisting of 2 hospitals, primary care, and partial home care. To gather different stakeholder perspectives, several roles related to the HF care process were represented, that is, medical process leaders, medical specialists in cardiology, specialist nurses, physiotherapist, home care physician, home care nurses, and controllers. The interviews involved identifying the roles that may have relevance for the implementation of the application. Thus, the stakeholder network grew organically by recommendations from interviewees during the interview period (4 months) and continued until no further representatives of perspectives or roles were identified by the participants.

### Data Collection

#### Overview

The interviews were semistructured and performed one-to-one using videoconferencing (due to the COVID-19 pandemic restrictions). The interviews were recorded using a voice recorder and covered individual stakeholder perspectives and organizational perspectives related to AI applications and technology in general and in relation to the specific case. The questions related to the following topics: stakeholder role and working process, relation to the discharge process, experience of technology and AI system implementation, possible effects of a readmission prediction system (on patient, professional, and organizational levels), and success factors and barriers for implementation.

#### Contextual Information

The context in which this study was performed is one where there is an outspoken ambition from the regional health care system to work strategically to be at the forefront of using data to accomplish more information-driven care with improved quality and safety of care and more optimized use of resources [14]. The clinical professionals participating in this study were only partially aware of the investments into infrastructure and research around the ambitions on developing approaches for information-driven care, which also manifested in the varying knowledge around AI applications and their potential in the health care setting among stakeholders. Further, the care organization was, at the moment of the study, implementing a care process improvement scheme for newly debuted patients with HF based on the national guidelines [15] for HF care, which had the goal of reducing the number of readmissions. The organization had also recently increased the capacity to follow up patients in the outpatient clinic to meet the criteria of a first patient follow-up within 1 week following a discharge for newly debuted patients with HF. However, they still struggled to identify which patients were the ones to be chosen for such care at the hospital rather than being referred to primary care.

### Data Analysis

Data analysis was based on transcribed interviews and sorted into themes such as the care process, potential organizational effects, and impact on users [16]. To further identify barriers and enablers to implementation of the AI application, the data were coded [17] as per the categories defined in the nonadoption, abandonment, scale-up, spread, sustainability (NASSS) framework (Table 1): the condition, technology, value proposition, adopter system, organization, wider system, and embedding and adaptation over time [18]. Two researchers performed the coding separately and were blinded from each other. If there was a conflict of agreement, a third researcher was involved to reach a consensus.

**Table 1:** Examples of data coding.

Insight	NASSS^a^ domain	NASSS part
1. AI^b^ tools (referring to self-monitoring tools) create extra work—introducing and supporting patients	Adopter system	4A
2. Patients contacting staff when AI tool does not work or provides no clear guidance	Adopter system	4A
3. Fear of gradual decrease in knowledge—AI replacing decision makers	Adopter system	4A
4. Positioning the AI tool as a support for decision-making, in combination with professional expertise	Adopter system	4A
5. A support function separate from clinical staff	Technology	2C
6. Extra human resources should be planned before introducing the solution: for providing support and for working with the identified patients	Organization	5D
7. Older age or generation can have difficulty getting used to working in a new way using AI	Technology	2A
8. Intuitive interface	Technology	2A
9. Introduction or implementation of AI solution should be led by a person with health care background with IT^c^ interest or skills	Organization	5E
10. The tool providing more nuanced information in the referral about the follow-up needs of a patient, for example, urgency	Technology	2B
11. Presenting factors for the risk of readmission	Technology	2B
12. Text in the warning for the risk of readmission should be carefully selected to be helpful in decision-making	Technology	2B
13. Affected roles: physicians, nurses, physiotherapists, and occupational therapists	Organization	5D

^a^NASSS: nonadoption, abandonment, scale-up, spread, sustainability.

^b^AI: artificial intelligence.

^c^IT: information technology.

### Ethics Approval

The parts of this project that are handling sensitive information and personal data are covered within the ethical approval by the Swedish ethical review authority (2022-07287-02). The participants in this study were given information about the study in writing when asked to participate and oral information prior to the interview along with consent to participate. There were no personal data or sensitive information collected in the material presented in this paper. All interviews were transcribed prior to data analysis to prevent any voice identification being available. No compensation was given to participants.

## Results

The different stakeholders shared views on the problem and complemented each other in bringing up different barriers and enablers for implementation.

### Domain 1: The Condition

The stakeholders highlighted that although the goal is to reduce the readmission rate, the readmissions, when necessary, save lives. One respondent highlighted, for example, that a patient getting proper treatment while being admitted can be discharged at a lower risk of being readmitted within days or weeks compared to someone who is sent home too early in the care process. However, stakeholders are also aware that not all patients can be prioritized for full treatment, which could mean additional days at the ward or referral to the outpatient clinic for quick and continuous follow-up. Thus, they acknowledge the need to identify patients at high risk of readmission to potentially avoid additional readmissions.

### Domain 2: The Technology

The stakeholders identified an intuitive interface as an important aspect of the sustainable adoption of an AI application. It could facilitate the use of the application by clinicians of all age groups since older age was identified as a potential barrier to sustainable adoption. In addition, the application should, in real time, consider critical events, different symptoms, treatments applied, and new test results that correlate with readmission. In addition to the risk level, factors that led to the conclusion should be presented as they would be helpful in the clinician’s decision-making. The application should also update the risk assessment based on up-to-date data every time a clinician opens a journal. When a patient with the risk level is admitted, a notification should reach all relevant units (eg, outpatient clinic, home care, or primary care). All these units should be able to access information on such a patient. The stakeholders feared being burdened by the technical issues if the system is disrupted or contains bugs, as it would cause extra workload but also potentially provide false results. Hence, a support function should be designated to help with technical issues for a smooth clinical workflow.

Another concern was to trust that appropriate and relevant data are included in the data set of the algorithm to inform the risk assessment. If the algorithm does not consider medical notes written free-style, relevant information would be missed. A checklist guiding further procedures including follow-up needs for the patient (eg, urgency) based on a received risk assessment could highly support the new workflow. Such procedures should be based on clinical guidelines and adapted to the standardized care path. The application should be interoperable with other information systems already existing in a health care organization.

### Domain 3: Value Proposition and Value Chain

The primary value of the applications is for the patients. Through timely follow-up and interventions, fewer readmissions would result in less suffering for the patients. Stakeholders highlighted this value by stating, for example, “This is what we are after...to have patients feeling well, that do not have to go in and out of hospital and suffer due to lack of proper treatment.” Further, the stakeholders also mentioned the economic value of reducing the number of total admissions. As one of the stakeholders said,

We reduce enormous suffering. We make their quality of life better at home, AND we can get an economic lift in our region. Every one of the days is expensive.

Another value of the application that was mentioned was the potential to remove subjectivity in the assessment of the risk of readmission, thereby increasing equality in the care received.

### Domain 4: Adopter System

The role of the clinical professionals was perceived to either remain unchanged or be boosted throughout the care process if the application were taken into routine use (although some mentioned that their knowledge and skills might gradually decrease if they only relied on the application). First, if the risk assessment was available early in the process of admission, the clinicians would feel more prepared to meet the specific needs of a patient. Second, an early indication of the risk level could trigger early collaboration with other units, which would allow for the possibility to adapt the referral procedure and medications based on the risk level. Third, resource and time allocation would be more purposeful—the risk assessment would allow prioritizing patients, scheduling an earlier return visit, and redirecting human efforts. Fourth, the aspect of liability for taking action should be thought through and set in procedures when the clinicians disagree with the risk level assessed by the application and take different actions than the risk estimate would indicate. The stakeholders concluded that the responsibility should remain with clinicians.

### Domain 5: Organization

**Figure 1:**
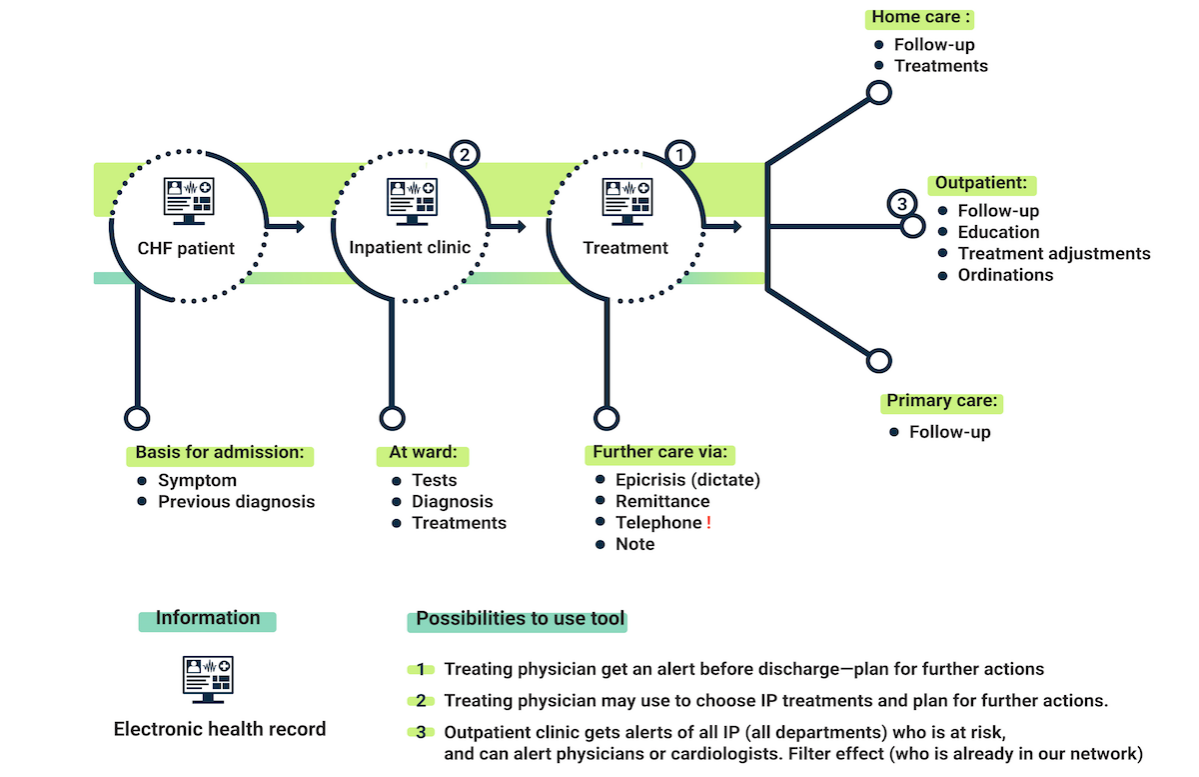
An overview of the patient pathway. The numbers 1, 2, and 3 represent the different places where stakeholders suggested the application could potentially be useful. CHF: congestive heart failure; IP: inpatient.

Besides using the application in the discharge process, clinicians saw opportunities to also use the application in the guidance of inpatient clinic treatments, that is, earlier in the care process (Figure 1). Then, the system needs to provide relevant information explaining the reason for the risk score so that the clinician can act upon the information. As one respondent said, “It is also important to see how the risk changes during the care process, when a treatment is prescribed.” Another use of the application could be in an outpatient clinic to guide in the identification of high-risk patients in need of urgent follow-up:

If we see that it is a patient with high risk, we can prioritize a visit to the HF clinic instead of sending the remittance to primary care. That should also allow for a quicker management.

However, they worried about the additional workload if too many patients are deemed at high risk for readmission within 30 days. Figure 1 provides an overview of the patient pathway and information basis from being admitted to the inpatient clinic, to the further care decided upon.

Variations in key performance indicators, end points, and measurement practices by different clinician roles might create tensions during the implementation of the application. For example, this issue might emerge when resources need to be relocated (eg, from inpatient care to outpatient care) potentially creating a risk of protectiveness and resistance to the adoption of the application.

The stakeholders pointed out that a pilot study involving the units that concern the HF treatment process could strengthen the case of adopting the application with the necessary evidence and increase trust in it. Such a study should compare staff experiences of using the application and outcomes against usual care. To increase support for using the application, introduction and implementation of the application should be led by persons with a health care background and with information technology interests or skills. A broad information campaign with involvement of related units needs to be organized—it could create responsibility for the results of using the application. Training should involve all relevant clinical professions and ensure that the algorithm is explained—understanding the solution would increase trust. Examples could be taken from other hospitals that have succeeded in using similar applications and discussing how to translate these experiences into their own context. Extra resources should be allocated to the implementation task, and the current responsibilities of staff should be revised to not create tensions due to overburdened staff.

The application would concern physicians, nurses, physiotherapists, and occupational therapists, among others. Further, use of the application should be mandatory and emphasized during staff meetings. To facilitate adoption and onboard new staff, time should be allocated for reflective activities during staff meetings, potentially leading to ideas on how using the application could improve the work. Furthermore, if the algorithm needs to consider data currently available in medical notes or requires additional data, clear routines would be required for data entry to ensure that the application makes decisions on good-quality data.

### Domain 6: Wider System

The stakeholders noted that political pressures to release patients and keep beds available (eg, as experienced through the COVID-19 pandemic) might bring additional factors into the decision-making process regarding patient discharge.

### Domain 7: Embedding and Adaptation Over Time

The market and new technologies entering health care and other disciplines will naturally push toward the need to implement new applications not only in cardiology but also in other specialist areas. Stakeholders mentioned, for example, the potential to apply a similar application for predicting readmission in the care of patients with chronic obstructive pulmonary disease. However, some respondents also highlighted the risk of “getting stuck” with applications and products that are not proven effective over time. Therefore, the abandonment of nonuseful applications should be practiced more as it leads to better motivation to accept new technologies by staff and reduces workload and fatigue caused by ineffective applications.

## Discussion

The study aimed to understand the context and stakeholder perspectives for the future implementation of an AI-based decision support application for predicting readmissions of patients with HF.

### Principal Results

The study showed that stakeholders see potential value in the AI-based decision support application for the prediction of readmissions and would like to see it integrated into routine practice. It also identified that stakeholders need decision support and risk assessment early in the care process—during admission or treatment follow-up (Figure 1), although the AI model was initially developed to be a support system used in the discharge process of a patient. The primary perceived value of the AI model was in reducing the uncertainty in caring for and discharging patients with HF. Additional value was seen through a potential increase in equality in the decisions concerning the patient. For that, there needs to be certainty behind the AI model itself: (1) the data considered should be relevant and all the relevant data should be included, (2) readmission risk levels should be based on clear thresholds derived from the scientific literature, and (3) factors that lead to a particular risk level should be displayed to the clinician in an understandable way. To sum up, the competence of the AI model should be monitorable and verifiable by clinical staff to allow them to fully oversee the result.

Although the results showed much perceived value in implementing the clinical decision support tool, there were several aspects brought up that need careful consideration. For example, stakeholders brought up the risk of the tool leading to an overload of prioritization needs. Such effects could lead to increased workload, for example, for the staff at the outpatient clinic, who follow up on urgent cases within a short period of time after discharge. Another important barrier is technology fatigue [19]. Clinicians were skeptical about including one more digital application in their daily practice since, in the case of failure, organizations are usually hesitant to abandon technologies that turn out to be ineffective, which adds to clinicians’ fatigue. Clinical validation being an increasingly emphasized and required activity for technology developers, clinicians’ fatigue is a critical aspect that could inhibit innovation and efforts to support and transform health care. Therefore, future research should explore what organizational set-ups and incentives could support the interest and willingness of clinicians to test similar solutions to create better chances of AI adoption in practice.

### Limitations

Some of the clinicians had little experience with AI systems, and this study reflects on feedback from potential users and provides input to the development of the system as well as planning of the implementation process. As stakeholders mentioned, the interface design and smooth functionality of the system are key components to be addressed before implementation can even be thought of. Therefore, this study needs to be followed up by design iterations with clinicians and other potential users to go more in detail on matters brought up in this study, which involves design of the AI model, design and integration of the system, and design of the implementation process.

The generalizability of these results may further be limited by contextual factors, such as the size of the region, the data infrastructure, and the participants’ prior knowledge and understanding of the impact of AI in health care. However, many aspects brought up by participating stakeholders align with previous studies in this area. One important thing to highlight, however, is that few of the stakeholders problematized the adoption of AI in health care other than mentioning issues such as the fear of gradually decreasing skills, liability aspects, and potential effects on, for example, resource allocation. Potentially, this could be an effect of how the decision support system was presented to the respondents, the nature of the questions, or the prior knowledge that these stakeholders had about the development projects related to information-driven care being ongoing in the region. Nevertheless, it is of importance to also consider problems in the integration of AI tools in practice when planning development, design, and implementation processes [20].

### Comparison With Prior Work

This study confirms that AI-based decision support tools to reduce the risk of readmission of patients with HF can primarily solve the problem of uncertainty for clinicians, which was a problem identified in previous studies [21,22]. However, there was a lack of understanding of where in the process and under which circumstances this uncertainty is the highest and most pressing. This research has added knowledge that such an AI model would be most useful not only during discharge but during the whole admission process of the patient, especially in care units that do not specialize in HF but have admitted a patient with HF for a different reason. Due to the discussions with the stakeholders and the identified clinical gaps, changing the fundamental structure and data sets of the machine learning algorithm may be required. In addition, input from stakeholders early in the process of technology development can prevent wasting resources on suboptimal applications that lack clinical relevance and add workload to clinicians while testing the application, as highlighted in previous research [23].

The liability concerns that were brought up by the stakeholders provide a pressing need and one more question to the ongoing liability discussion [24,25]: Who is to be held responsible when the clinician disagrees with the application’s outcomes and takes a different course of action that results in an adverse event? Because the system maintains the record of the system’s suggestion, the liability when basing decisions on own expertise that goes against the AI model becomes not so trivial. These findings are in line with the discussion on liability and the “responsibility gap” dilemma when it comes to AI-based clinical decision support applications [26] and can contribute to the discussion on potential solutions for liability issues, such as AI liability insurance [27].

### Conclusions

An AI-based decision support system for assessing the readmission of patients with HF can provide value in helping to prioritize patients, reducing uncertainty in decisions and coordinating work among care units. Such a system can provide the best value if used throughout the admission process rather than only at discharge. For such a system to positively impact patient care, innovation and implementation aspects need to be carefully considered in light of the stakeholders affected. Specifically, the barriers and enablers for implementation of a clinical decision support system predicting risk of readmission are related to the seven domains of the NASSS framework: (1) the condition—there is a clear need for such a system; however, there are aspects in relation to the primary use of the system, for example, prioritization and treatment plans, that needs careful consideration; (2) the technology—that the system is designed to be user friendly and useful, that relevant data are considered by the AI model, that data are updated in real time, that access is available to the system information at any time, and that there is integration to standardized routines; (3) the value proposition—a reduced burden on patients and economic cost savings; (4) the adopter system—additional information about the patient increases the ability to make decisions and reduces liability issues when there is a conflicting decision made that results in an adverse event; (5) the organization—how different people may use the system, where in the care process it should be used, and how the system output may change the flow of patients, thus affecting the resources needed at different areas of the organization; (6) the wider system—for example, political pressures on resource use during crisis; and (7) embedding and adaptation over time—for example, the risk of being “forced” to use a system if it does not live up to the purposes or gets outdated.

This study further highlighted the importance to study stakeholder needs early in the process of development, design, and implementation of decision support systems and to be prepared to restructure the project based on insights from stakeholders.

## Abbreviations

AI: artificial intelligence
HF: heart failure
NASSS: nonadoption, abandonment, scale-up, spread, sustainability

## Data Availability

The data sets generated during and/or analyzed during this study are available from the corresponding author on reasonable request.
